# Local Long-Term Inner Ear Drug Delivery in Normal Hearing Guinea Pig—An Animal Model to Develop Preventive Treatment for Noise-Induced Hearing Loss

**DOI:** 10.3390/biom12101427

**Published:** 2022-10-05

**Authors:** Kathrin Malfeld, Peter Baumhoff, Holger A. Volk, Thomas Lenarz, Verena Scheper

**Affiliations:** 1 Department of Otolaryngology, Hannover Medical School, Carl-Neuberg-Str. 1, 30625 Hannover, Germany; 2 Department of Small Animal Medicine and Surgery, University of Veterinary Medicine Hannover, Foundation, 30559 Hannover, Germany; 3 Institute of AudioNeuroTechnology and Department of Experimental Otology, ENT Clinics, Stadtfelddamm 34, Hannover Medical School, 30625 Hannover, Germany; 4 Cluster of Excellence “Hearing4all”, German Research Foundation (DFG; “Deutsche Forschungsgemeinschaft”), Hannover Medical School, Carl-Neuberg-Str. 1, 30625 Hannover, Germany

**Keywords:** osmotic pump, local drug delivery, cochlear pharmacotherapy, temporary threshold shift, prevention

## Abstract

Noise-induced hearing loss (NIHL) is one of the leading causes of sensorineural hearing loss with global importance. The current treatment of choice for patients with hearing problems is a hearing aid or a cochlear implant. However, there is currently no treatment to restore physiological hearing. The development of preventive drugs is currently the focus of hearing research. In order to test the efficacy of a drug, the active ingredient has to be applied at reliable concentrations over a period of time. Osmotic minipumps can provide local drug delivery into the perilymph. Combined with a cochlear implant or a tube, the implantation of the pumps may lead to increased hearing thresholds. Such surgery-related threshold shifts complicate the examination of other factors, such as noise. The aim of the present study was to develop an animal model for the examination of substances that potentially prevent NIHL. For this purpose, six male guinea pigs were unilaterally implanted with a silicon catheter with a hook-shaped microcannula at its tip, attached to an artificial perilymph containing osmotic minipump. One week after surgery, the animals were exposed to four hours of a musical piece, presented at 120 dB SPL, to induce a threshold shift. The implantation of the hook-delivery device caused a moderate threshold shift that allows to detect an additional noise-induced temporary threshold shift. This method enables to investigate drug effects delivered prior to the noise insult in order to establish a preventive strategy against noise-induced temporary threshold shifts. The established drug delivery approach allows the release of drugs into the inner ear in a known concentration and for a known duration. This provides a scientific tool for basic research on drug effects in normal hearing animals.

## 1. Introduction

Excessive overexposure to noise due to occupational or recreational activities can lead to noise-induced hearing loss (NIHL), one of the leading causes of sensorineural hearing loss (SNHL) in people. SNHL is characterized by hearing threshold elevation, caused by a loss of cochlear sensory cells and a subsequent degeneration of the spiral ganglion neurons (SGN) and their central projections [1,2,3,4,5,6,7]. Moreover, noise trauma of the ribbon synapses between the hair cells and the peripheral neurites of the SGN can lead to a synaptopathy, which has been identified to cause “hidden hearing loss” [8,9,10,11]. Patients with this type of SNHL show a reduction in wave I amplitude in the acoustically evoked auditory brainstem response (ABR), but no threshold shift. The patients do struggle with their hearing, especially in noisy environments [12]. Globally, 16% of hearing loss in adults is attributed to work-related noise exposure. Employees in a wide range of occupations are exposed to potentially harmful levels of noise, either from one-time exposure to impulse noise or by continuous ambient exposure [13]. Aside from occupational exposure, approximately 5% of the world’s population suffers from NIHL and 1.1 billion adolescents and young adults are at risk of noise-related hearing loss [14,15]. Unfortunately, no therapy of NIHL has been established, except for hearing aids or cochlea implants (CIs). Therefore, the prevention of NIHL is currently the focus of research. The outcome of conventional prevention methods, such as earplugs or other hearing protection devices, varies depending on user compliance. Additionally, the use of protection devices is not always possible in work situations. In order to circumvent these problems, one concept is the pharmacological prevention of NIHL, which is currently being investigated in several animal and some human studies [16,17,18], including soldiers. Preventive strategies that are under investigation include antioxidants [19,20], glucose [21], neurotrophic factors [22,23], vasodilators [24], glutamate antagonists [25] and anti-inflammatory agents [26]. Whereas the noise exposure of healthy humans leads to ethical conflicts, most of the studies that address the development of preventive strategies against NIHL involve animal experiments.

Guinea pigs are a commonly used animal model in hearing research. They are used to assess the normal structure and function of the cochlea and auditory pathways, as well as for studies on therapy development for the pathological auditory system, after damage induced by noise exposure, ototoxic drug treatment, or other insults [27]. Development of pharmacotherapies for inner ear treatment is one major research area that utilizes guinea pigs as an animal model. Previous studies that investigated preventive treatment used various concepts for drug delivery, such as the application of gel matrices [23,28] or a bolus injection [29] on the round window or injection of the substance intraperitoneally [21,30]. However, in order to understand how a drug affects the cell function, the delivery of the active ingredient must be reliable in terms of the concentrations achieved at the target side. Local fluid-based delivery into the perilymph allows the application of a precise drug concentration, without the release kinetics being affected by tissue boundaries between the delivery device and the target cells or a delivery matrix with unknown drug release kinetics. Osmotic pumps are well-established systems for continuous drug delivery in hearing research [31]. They can be combined with a CI [32,33,34] or a tube [22,31,35,36], which are inserted into the cochlea via the round window or a cochleostomy [22,37] for local drug delivery. The insertion of the CI or tube tip may lead to increased hearing thresholds in the normal hearing animal model [38], comparable to residual hearing loss in CI patients. These surgery-related threshold shifts can be caused by the surgical procedure itself or by implant-associated factors, such as foreign body reactions or dislocation of the electrode [39]. Aside from that, even the application of artificial perilymph to the intact round window membrane may cause increases in hearing threshold [40]. Since the investigation of strategies against NIHL needs a normal baseline threshold, the impact of surgery-related threshold shifts sometimes leads to exclusion of animals from studies that aim to investigate the preventive effect of a substance against NIHL.

The aim of the present study was to develop a pump-catheter system for chronic drug delivery in an animal model with low surgery-related threshold shifts, which allows the investigation of the effect of subsequent noise trauma and the future treatment strategies against NIHL.

## 2. Materials and Methods

### 2.1. Pump Preparation

**Figure 1:**
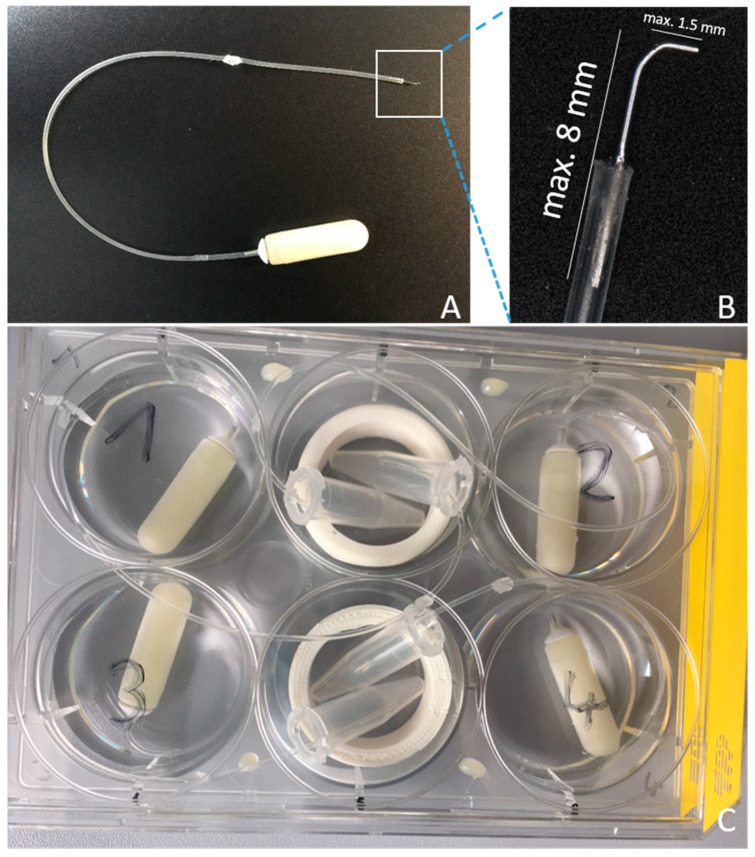
The self-build hook-delivery device (HDD). (**A**) The HDD consists of an ALZET^®^ rat jugular catheter and a small microneedle (Nordson Optimum^®^) attached to an osmotic pump (ALZET^®^ 2006). (**B**) Close-up view of the catheter’s tip. The maximum length of the microneedle after bending was 8 mm and for the shank inserted in the scala tympani, it was 1.5 mm. (**C**) Incubation of the pump with attached catheter in a 6-well plate prior to implantation. For quality control, the induced pumping was checked visually by inspection of fluid filling of Eppendorf^®^ tubes.

A commercially available silicone catheter (ALZET^®^ rat jugular catheter, DURECT Corporation, Cupertino, CA, USA; 0.94 mm OD; 0.51 mm ID) was combined with a small stainless-steel tip (Nordson Optimum^®^ #7018433, Nordson Deutschland GmbH, Erkrath, Germany), with an outer diameter of 0.31 mm. For this purpose, the cone was removed and the cannula was bent to an angle around 90°. The shorter shank had a maximum length of 1.5 mm. The longer shank was shortened to a maximum length of 8 mm and inserted in the catheter. The catheter and tip were connected using tissue glue (Indermil^®^ flexifuze™, Connexicon Medical, Dublin, Ireland), paying attention to not block the lumen (Figure 1A,B). After the adhesive was fully cured, the construction was tested for permeability by injecting sterile 0.9% saline (B.Braun, Melsungen, Germany) to control fluid drain off at the tip macroscopically. The liquid was completely removed using an air-filled syringe and the catheter was irradiated with UV light (Spectrolinker™ XL-1000 UV crosslinker, Spectronics Corporation, Westburry, NY, USA) for 30 min to eliminate microbiological contamination.

To evaluate the impact of the implantation of a local drug application system on the hearing threshold, we used artificial perilymph (AP; 145 mM NaCl, 2.7 mM KCl, 2.0 mM MgSO_4_, 1.2 mM CaCl_2_, 5.0 mM HEPES, pH = 7.4 with 0.1% guinea pig serum albumin) [27] as the model fluid. Filling of the osmotic pumps (ALZET^®^ 2006, DURECT Corporation, Cupertino, CA, USA; pumping rate 0.15 µL/h) and catheters was conducted under sterile conditions, with a flow based on the guidelines of the manufacturer. The self-made hook-delivery device (HDD) was attached to the pump flow moderator by insertion. To avoid disconnection of the catheter and flow moderator, a small drop of UV cement (Tetric EvoFlow^©^, Ivoclar Vivadent, Schaan, Liechtenstein) was used. Since a catheter was used, additional priming of 60 h was needed, as required by the manufacturer. During this time, the pumps were placed in a 6-well-plate (TPP^®^ #92006, Trasadingen, Switzerland), with every tip of the catheters positioned in a 0.5 mL Eppendorf^®^ tube (Eppendorf AG, Hamburg, Germany) (Figure 1C). This enables the observation of liquid coming out of the catheters tip during the priming time. Pumps were covered with 0.9% saline (B.Braun, Melsungen, Germany) and the plate was stored in an incubator at 37 °C and 5% CO_2_.

After the in vivo tests (see below), the pumping of the explanted pumps was re-checked using the same procedure.

### 2.2. Animals and Experimental Timeline

Six adult male Dunkin-Hartley guinea pigs (Charles River Laboratories, Châtillon, France), weighing between 374 g and 426 g, were used. All animals received an osmotic pump implanted unilaterally in the left ear (n = 6 implanted ears, n = 6 right not implanted ears) and all ears were exposed to noise trauma (n = 12 implanted and not implanted ears exposed to noise). They were kept in a temperature- and humidity- controlled room, exposed to a 24-h light–dark cycle (14 h/10 h), with free access to food and water. All experimental procedures were conducted in accordance with the German “Law on Protecting Animals” and with the European Communities Council Directive 2010/63/EU for the protection of animals used for experimental purposes. The use of animals for scientific purposes was permitted by the local authorities (Lower Saxony State Office for Consumer Protection and Food Safety (LAVES), Oldenburg, Germany, registration number 19/3145).

**Figure 2:**
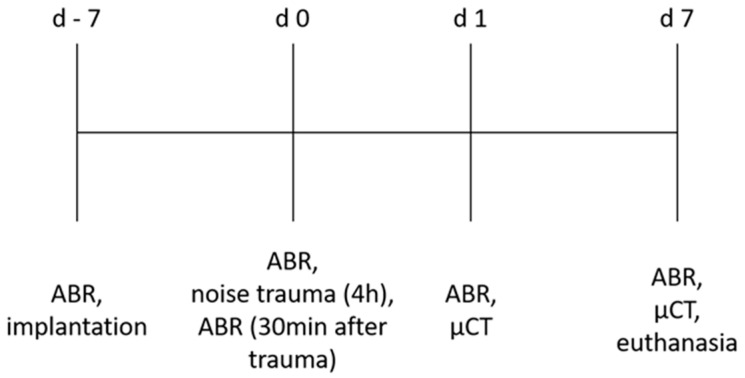
Experimental set-up. Timeline of the individual measurements and interventions.

Initially, the animals’ normal hearing was verified by ABR measurement on day −7. Subsequently, the animals were implanted with the HDD. After 1 week, on day 0, an additional ABR was performed, followed by four hours of noise exposure. In addition, 30 min after noise application, the hearing status was measured again. One day after noise insult (day 1), an ABR measurement, followed by imaging of the drug delivering device in situ using µCT, was performed. On day 7 after noise insult, a final ABR was performed, followed by µCT imaging and euthanasia. This timeline is illustrated in Figure 2.

ABR measurement, HDD implantation, noise trauma, µCT and euthanasia were performed under general anesthesia (intramuscular medetomidinhydrochloride 0.2 mg/kg, midazolam 1 mg/kg and fentanyl 0.025 mg/kg), following pre-anesthetic sedation (oral diazepam 4 mg/kg). Areas to be incised were locally infiltrated with prilocaine. To reduce pain and to prevent infections, the animals subcutaneously received 0.2 mg/kg meloxicam and 10 mg/kg enrofloxacin. Euthanasia was performed via intracardiac injection of no less than 300 mg/kg pentobarbital. For details concerning the medical treatment, one can refer to Appendix A.

### 2.3. Acoustically Evoked Auditory Brainstem Response (ABR) Measurement

**Figure 3:**
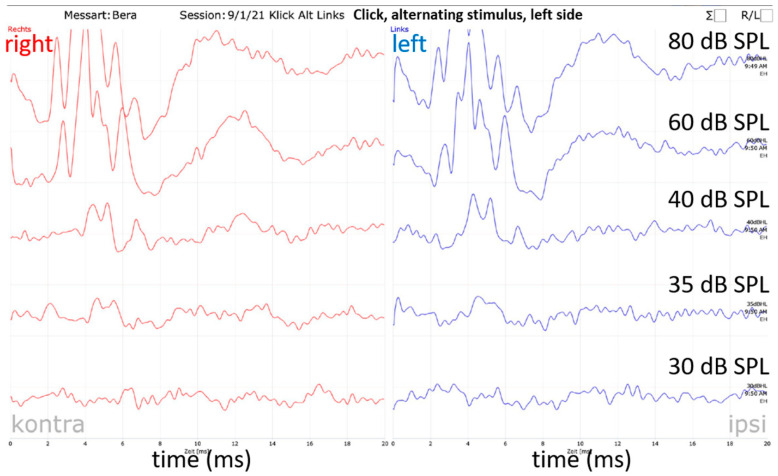
Hearing threshold determination. The visual hearing threshold determination exemplified by a naïve (d −7) click measurement of the left side. It is a printout of the software’s evaluation file. Blue curves show the left-side recordings (stimulated side) and red curves the right-side recordings. Since there is no clear ABR signal on the left side below 35 dB, the hearing threshold was set at 35 dB SPL.

Acoustic stimulation and recording of the auditory brainstem signals were performed using a Pilot Blankenfelde system modified for use in guinea pigs (Pilot Blankenfeld Medizinisch–Elektronische Geräte GmbH, Blankenfelde-Mahlow, Germany). The ABR signals were recorded using four subdermal needle electrodes. They were placed at the vertex (common positive), left and right mastoid (references) and in the neck (ground). Due to the implants’ position and the head suture, the vertex and neck electrodes’ position had to be changed from day 0. The positive electrode was placed rostral to the head suture and the ground electrode caudal to the osmotic pump. Experiments were conducted in a soundproof booth. To detect general auditory thresholds, acoustic clicks (duration 150 µs) were used. For detection of frequency-specific acoustic thresholds tone bursts of 500 Hz, 1 kHz, 2 kHz, 4 kHz, 8 kHz, 16 kHz, 32 kHz and 40 kHz were used. Acoustic stimuli were presented by loudspeakers (ER•3C™, Etymotic Research, Inc.,Elk Grove Village, IL, USA for click and ≤ 4 kHz; EC 1, TDT, Alachua, FL, USA for ≥ 8 kHz) via a plastic cone placed in the outer ear canal. The EC1 gained electrical supply via an electrostatic speaker driver (ED 1, TDT, Alachua, FL, USA). All loudspeaker-cone compositions were calibrated at the beginning of the project. For this purpose, the system’s output was adjusted in the service menu of the software at 80 dB for each stimulus. The output was recorded using a ¼-inch condenser microphone (type 4939, Brüel & Kjaer, Nærum, Denmark) connected to a preamplifier (type 2670, Brüel&Kjaer, Nærum, Denmark) and a conditioning amplifier (type 2690, Nexus conditioning amplifier, Brüel & Kjaer, Nærum, Denmark). Before the end of the study, the output was tested again and was 82.06 ± 5.73 dB SPL. Starting at 80 dB SPL (or peak equivalent for click stimulation), each stimulus was presented 200 times and responses from the contralateral ear were masked by white noise 30 dB below the stimulus level. Dependent on the response, the hearing threshold was searched down- or upwards in 20 dB to 5 dB steps. The hearing thresholds were determined by visual inspection of ABR signals in the analyze function of the system, with a maximum magnification of 700 nV/Div. The lowest stimulus intensity at which ABR signals could be detected was taken to be the hearing threshold for the relevant stimulus configuration (Figure 3). Where the hearing threshold could not be identified up to the maximum sound stimulus level of 100 dB SPL (85 dB SPL for 40 kHz), the threshold was defined as 110 dB SPL or 95 dB SPL for 40 kHz, respectively. Only animals with initial normal hearing (click thresholds ≤ 40 dB SPL) [41] were included in the study. All animals fulfilled this criterion. To reduce the anesthesia time, frequency-specific thresholds at the right (not implanted) ear were measured only on day 0 and day 7. For a detailed overview of the performed ABR measurements, one can refer to Table 1.

The hearing threshold shift due to implantation was set as the difference between the measured thresholds of day −7 and day 0 pre noise exposure. The threshold shifts due to noise at different time points after exposure were set as the differences between the hearing thresholds of day 0, 30 min post noise, day 1, and day 7 in comparison to the day 0 pre noise threshold.

### 2.4. HDD Implantation

The anesthetized animal was placed on a heating pad and the skin over the skull was incised after local anesthesia with prilocaine. The periosteum was removed and a subcutaneous pocket between the scapulae was formed. Access to the middle ear cavity was obtained using a retroauricular approach. After visualizing the round window, the bony overhang of the round window niche was removed using a micro hook, without hurting the facial nerve. A subcutaneous tunnel that connected the skin incision at the skull and the postauricular incision was built, in which the catheter was guided to the middle ear cavity. The catheter tip was inserted in the round window after opening the membrane and the bulla was closed using Tetric EvoFlow^©^. The osmotic pump was placed in the subcutaneous pocket in the neck and the rest of the catheter was looped and placed on the animal’s head. To avoid tension stress on the implant tip, e.g., by head movement, a small piece (maximum length 7 mm) of a halved silicon tube (OD 4 mm) was fixed at the head using Tetric EvoFlow^©^ to guide the catheter. Additionally, a small drop of Tetric EvoFlow^©^ secured the catheter at the skull directly behind the subcutaneous tunnel. The postauricular wound was closed in two layers and the wound at the skull was closed with u-sutures.

### 2.5. Noise Trauma

**Figure 4:**
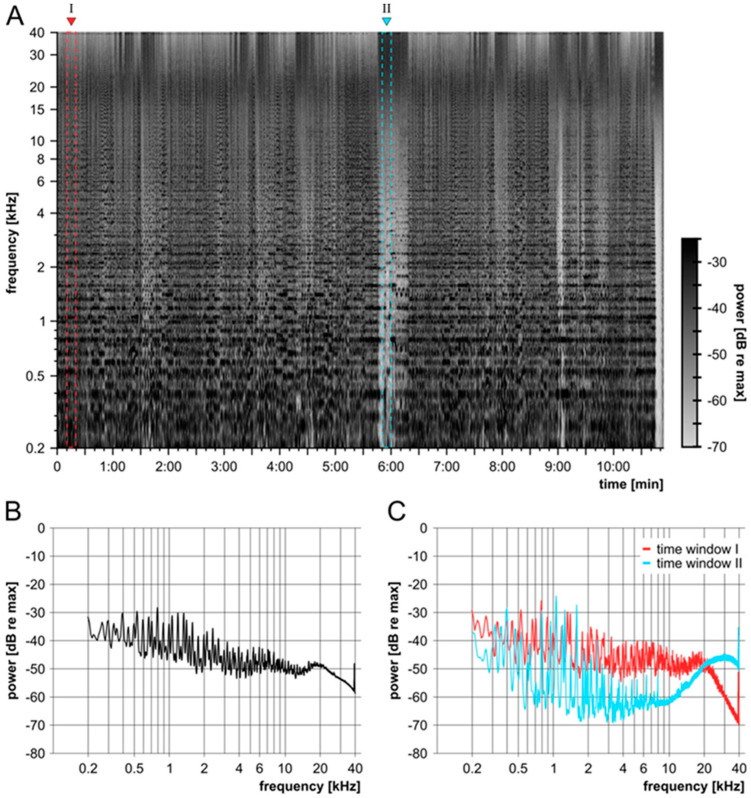
Description of the noise trauma. Characterization of the sound file for noise traumatization using Audacity^®^, illustrating the applied frequency range and intensity. (**A**) Spectrogram of the sound file that contains frequencies from 80 Hz to 48 kHz. The colored fields (red, I; blue II) indicate the time windows that are analyzed in C. (**B**) Mean spectrum over the whole time (10′54″) of the sound file. The maximum range between frequencies is 30 dB. (**C**) Spectra of two time windows of 10″. Time window I is located at the beginning (0′10″) and time window II in the middle (5′50″) of the signal. The spectrogram (A) was created using a Blackmann–Harris window with a width of 8192 samples. All spectra (B + C) were created using a Blackmann–Harris window with a width of 65,536 samples.

The anesthetized animal was placed on a heating pad in a sound-insulated box. Calibrated loudspeakers (DT48, 5 Ω, BeyerDynamic, Heilbronn, Germany) were placed directly in front of the animal’s outer ears. The noise insult was presented via the software Audacity^®^ (version 2.1.1) on a notebook (Windows 10 pro). Audacity^®^ software is copyrighted (^©^ 1999–2016 Audacity Team (Web site: http://audacityteam.org/, accessed on 2 June 2022)). It is a free software distributed under the terms of the GNU General Public License. The name Audacity^®^ is a registered trademark of Dominic Mazzoni. A stereo power amplifier (SA 1, TDT, Alachua, FL, USA) was interconnected between the computer and loudspeaker. The presented sound file was engineered for an overall broad, noise-like frequency spectrum beyond 40 kHz. To mimic naturally occurring fluctuations of the temporal envelope in noisy environments, the modified file was based on a recording of Beethoven 5th Symphony, 4th movement, Allegro; Presto, played by the Ensemble Reflector by PASCHENRecords. In order to create a broadband noise trauma, the audio file was limited in its dynamics, so that it had a constant digital level. In a second step, self-learning filters were used to generate a flat power spectrum between 200Hz and 40 kHz (maximum range was 30 dB between frequencies). The recording had a sampling frequency of 96 kHz and the sound file included all frequencies played by the instruments and inserted frequency content up to the Nyquist frequency of 48 kHz. Thus, a large part of the frequency range of the guinea pigs that extended above 50 kHz was excited by the stimulus [27]. The sound file had a length of 10 min and 54 s and was looped for 4 h. It was presented at 120 dB SPL, measured as the peak equivalent over the full frequency spectrum (Figure 4). The potential speaker output of the DT48 was above 100 dB SPL for frequencies between 800 Hz and 40 kHz.

### 2.6. µCT

For evaluation of the implants’ position, the cochlea of each guinea pig was scanned at day 1 and day 7 immediately after the ABR measurement under the same anesthesia using a µCT scanner (XtremeCTII, ScancoMedical AG, Brüttisellen, Switzerland). Scans were performed using 1470 µA, 100 W, at an integration time of 90 ms, resulting in a resolution of 17 µm. The data were converted to DICOM and reconstructed with COMET [42] and the position of the HDD in the round window was analyzed visually.

### 2.7. Statistical Analysis

Due to the small number of animals, data were double checked for normal distribution using the Kolmogorov–Smirnov test (in GraphPad Prism^®^ version 8.4.3.) and the Shapiro–Wilk test (at statskingdom.com) [43]. The Kolmogorov–Smirnov test reported all data sets to be normally distributed. Using the Shapiro–Wilk test, it was found that some data sets were not normally distributed. Click and frequency-specific hearing thresholds or threshold shifts over time within one experimental group and between left (implanted) and right (not implanted) ears were analyzed using GraphPad Prism^®^. For comparison of the groups with significant departure from normality, a Wilcoxon signed-rank test was used. If both data sets were normally distributed, a paired t-test was performed. For details concerning the statistical analysis, one can refer to Supplement File S1. The data are reported as mean ± standard deviation (SD). Statistical significance was considered and depicted at *p* ≤ 0.05 (*); *p* ≤ 0.01 (**) and *p* ≤ 0.001 (***). Data that did not significantly differ are indicated using ns = not significant.

## 3. Results

The main objective of the present study was to establish an animal model to test intracochlear preventive pharmacotherapy of temporary threshold shifts (TTS). A custom-made catheter-pump-based delivery system, allowing chronic drug delivery and imaging of the intracochlear part for quality control, was built, a TTS was established and the combination of both was tested in vivo.

### 3.1. Qualitiy Control of the Implant

**Figure 5:**
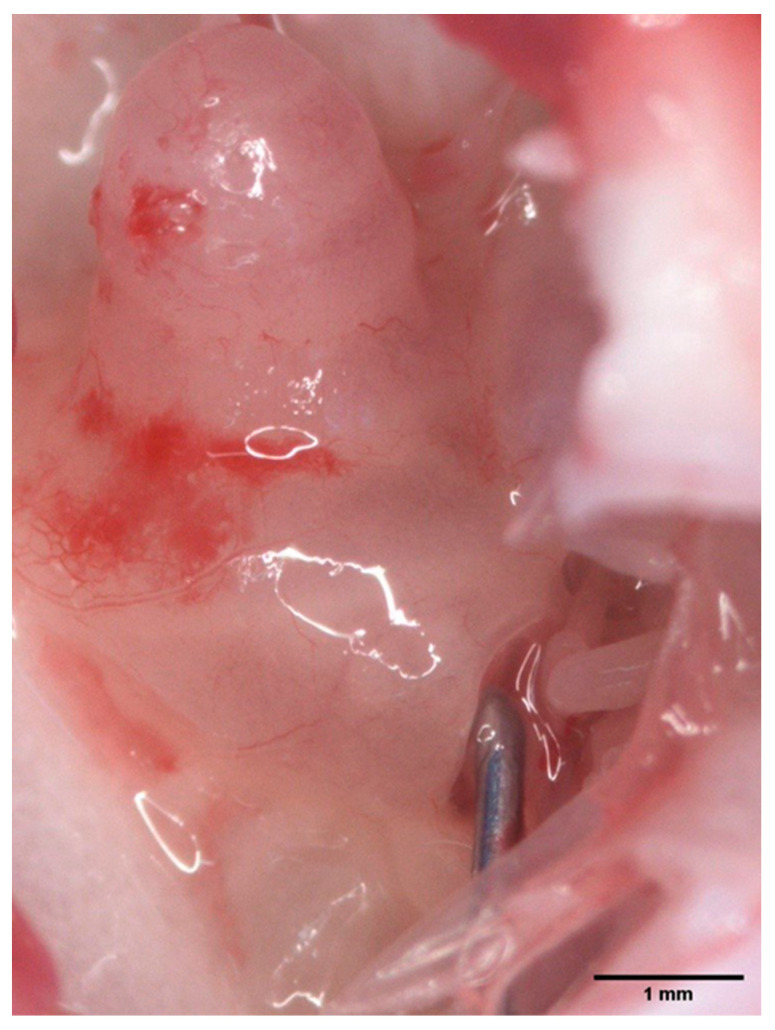
Implanted HDD. Image showing the HDD’s position in the round window. This was investigated macroscopically after euthanasia of the animal and harvesting the bulla. Scale bar indicates 1mm.

The self-built HDD was implantable into the guinea pig inner ear via the round window and remained in situ throughout the observation period. This was determined macroscopically after euthanasia of the animals (Figure 5), as well as via µCT (see Section 3.3 for details).

Before and after implantation, the fluid-filled delivery device was placed in a well plate to check pumping activity. This quality control method is an easy and inexpensive method to determine the functionality of each self-made HDD system. Before and after explantation, all pumps delivered fluid into the Eppendorf tubes, indicating proper function.

### 3.2. ABR Measurements

The study aims to determine both the effect of the HDD implantation and the effect of the noise trauma on the hearing threshold. To obtain an overview, first, the click hearing thresholds are reported, followed by the effects of the HDD implantation on frequency-specific ABR thresholds. Afterwards, the effect of noise exposure on frequency-specific ABR thresholds of the right (not implanted) ears was examined, followed by the effect of implantation and noise exposure on frequency-specific ABR thresholds of the left (implanted) ears. Finally, the effect of the implantation on the extent of the noise trauma was evaluated.

#### 3.2.1. Comparison of the Click Thresholds at Specific Time Points and Click Thresholds over Time

**Figure 6:**
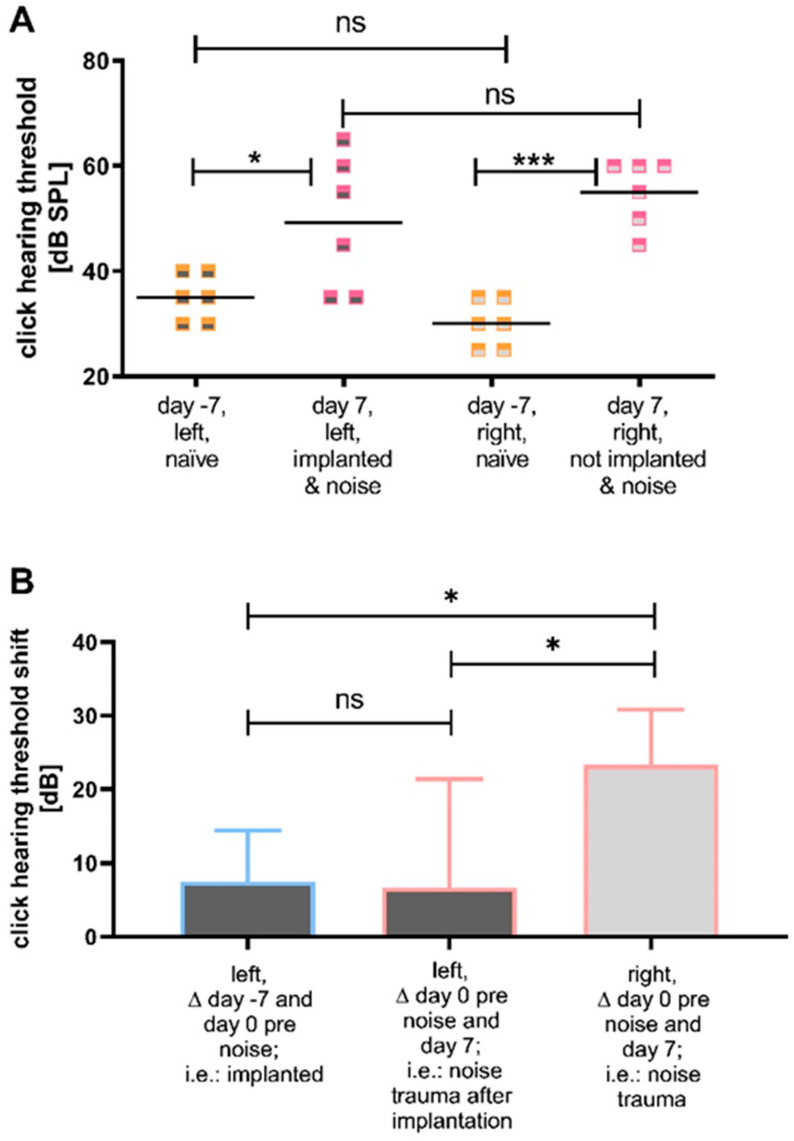
Implantation and noise exposure significantly increase ABR click thresholds. (**A**) The click hearing thresholds of both groups (left (implanted) (dark grey) and right (not implanted) (light grey)) when included in the study (day −7, naïve, orange) and 7 days after noise (pink) are illustrated. In both groups, a significant threshold increase is observed with a larger before–after difference in the not implanted ears than in the implanted ears. One week after noise, the hearing threshold of the two groups did not differ significantly. (**B**) The click threshold shifts of left (implanted) and right (not implanted) ears under different conditions. There is no significant difference between the click threshold shift in the left (implanted) ears from day −7 to day 0 pre noise (blue), compared to the threshold shift of the left (implanted) ears from day 0 before noise to day 7 (pink). Right (not implanted) ears show a significant difference in the threshold shift between day 0 pre noise and day 7, when compared to the shift of left (implanted) ears between day −7 and day 0 pre noise and between day 0 pre noise and day 7. ns = not significant; * *p* ≤ 0.05; *** *p* ≤ 0.001.

The initial naïve click-evoked hearing thresholds of left (pre-implantation) and right (not implanted) ears did not differ (Figure 6A). The click-evoked hearing thresholds of the left (implanted) and right (not implanted) noise traumatized ears did not differ 7 days after noise insult (Figure 6A). However, the click-evoked hearing thresholds on day −7 (naïve, before implantation and noise trauma) and day 7 (i.e., 7 days after noise trauma) differed significantly with 35 ± 4.4 dB SPL versus (vs.) 49 ± 12.8 dB SPL (*p* ≤ 0.05; paired t-test) for the left (implanted) ears, respectively, and 30 ± 4.4 dB SPL vs. 55 ± 6.3 dB SPL (*p* ≤ 0.001; paired t-test) for the right (not implanted) ears, respectively (Figure 6A). The comparison of the threshold shifts of the click hearing thresholds revealed a significant difference in the threshold shifts of left (implanted) and right (not implanted) ears between day 0 pre noise and day 7 (6.67 ± 14.72 dB SPL vs. 23.33 ± 7.53 dB SPL; *p* ≤ 0.05; paired t-test) (Figure 6B). Additionally, the shift of the click hearing threshold of the left (implanted) ears between day −7 and day 0 before noise differed significantly from the shift of the right (not implanted) ears between day 0 pre noise and day 7 (7.5 ± 6.98 dB vs. 23.33 ± 7.53 dB; *p* ≤ 0.05; paired t-test) (Figure 6B). The threshold shifts of the left (implanted) ears between day −7 (pre implantation) and day 0 before noise exposure did not differ significantly from the shift between day 0 before noise and day 7 (7.5 ± 6.98 dB vs. 6.67 ± 14.72 dB; paired *t*-test).

**Figure 7:**
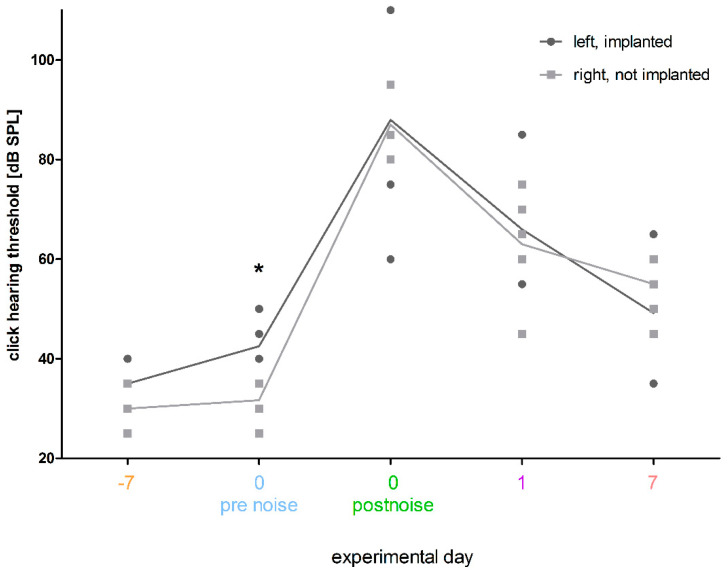
Effect of implantation and noise on ABR click thresholds over time. The mean (line) and individual (points) click hearing thresholds of left (implanted) (dark grey) and right (not implanted) (light grey) ears are illustrated over time. Since overlaps occur, the number of points does not correspond to the number of animals. There is a significant difference between the two groups on day 0 before noise trauma, i.e., 7 days after HDD implantation with left (implanted) ears showing a higher threshold. All other time points show no significant difference in the click hearing thresholds between groups. * *p* ≤ 0.05.

As the threshold increase during the whole experiment is more pronounced in the right (not implanted) ears than in the left (implanted) ears, the click hearing thresholds between the groups over time were compared (Figure 7) to elucidate a possible difference between the groups over time. On day 0, before noise insult, the thresholds of the left (implanted) ears were significantly increased compared to the right (not implanted) ears (42 ± 6.8 dB SPL vs. 31 ± 4.0 dB SPL; *p* ≤ 0.05; paired t-test). This difference could no longer be observed directly after noise trauma nor up to 7 days after noise trauma (Figure 7).

#### 3.2.2. Effect of HDD Implantation on Frequency-Specific ABR Thresholds

As the implantation appears to have an effect on the click-evoked hearing threshold that is neutralized after noise exposure, an analysis of the frequency-specific hearing thresholds was performed to look into the effect of implantation on the hearing function.

**Figure 8:**
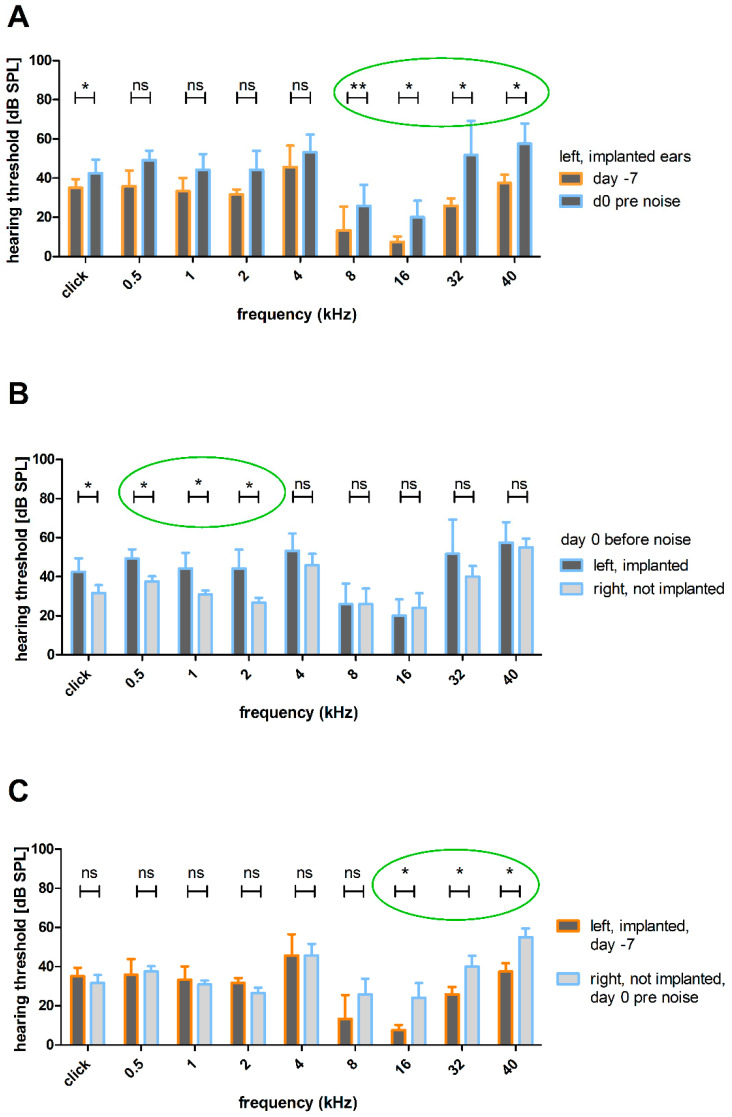
Effect of the HDD implantation on ABR thresholds. (**A**) Mean click and frequency-specific hearing thresholds of the left (implanted) ears before noise trauma compared to naïve condition. The implantation increased the threshold significantly in frequencies above 8 kHz (green circle) and also in the click condition. (**B**) Mean click and frequency-specific hearing thresholds of left (implanted) ears compared to right (not implanted) ones on day 0 before noise trauma. In contrast to the previously reported impact of the implantation (A), there is only a significant difference between left (implanted) and right (not implanted) ears in frequencies below 4 kHz (green circle) and in the click condition. (**C**) Mean click and frequency-specific hearing thresholds of left (implanted) ears on day −7 and right (not implanted) ears on day 0 before noise. There is a significant difference in frequencies above 16 kHz (green circle). ns = not significant; * *p* ≤ 0.05; ** *p* ≤ 0.01.

An increase in click-evoked and frequency-specific hearing thresholds was observed following the implantation of the HDD that delivered AP into the scala tympani. Thresholds of the left (implanted) ears before noise trauma (day 0 pre noise) were significantly higher at frequencies above 8 kHz than naïve ears (day −7) (Figure 8A). Compared to the right (not implanted) ears, the hearing thresholds of the left (implanted) ears do not differ on day 0 before noise in the middle and higher frequencies 4, 8, 16, 32 and 40 kHz (Figure 8B). However, at 2 kHz (44 ± 9.7 dB SPL vs. 26 ± 2.5 dB SPL; *p* < 0.05; Wilcoxon signed-rank test) and 1 kHz (44 ± 8.0 dB SPL vs. 30 ± 2.0 dB SPL; *p* < 0.05; Wilcoxon signed-rank test) and at 500 Hz (49 ± 4.9 dB SPL vs. 37 ± 2.7 SPL; *p* < 0.05; Wilcoxon signed-rank test) and in the click condition (42 ± 6.8 dB SPL vs. 31 ±- 4.0 dB SPL; *p* < 0.05; paired *t*-test), the thresholds of left (implanted) ears were significantly increased compared to the right (not implanted) ones (Figure 8B). In addition, the comparison of left (implanted) ears on day −7 and right (not implanted) ears on day 0 before noise exposure revealed significant differences in the higher frequencies 16 kHz (7.5 ± 2.74 dB SPL vs. 24.17 ± 7.36 dB SPL; *p* < 0.05; Wilcoxon signed-rank test), 32 kHz (25.83 ± 3.76 dB SPL vs. 40 ± 5.48 dB SPL; *p* < 0.05; Wilcoxon signed-rank test) and 40 kHz (37.5 ± 4.18 dB SPL vs. 55 ± 4.47 dB SPL; *p* < 0.05; Wilcoxon signed-rank test) with higher thresholds in the right (not implanted) ears (Figure 8C).

#### 3.2.3. Effect of Noise Exposure on Frequency-Specific ABR Thresholds

**Figure 9:**
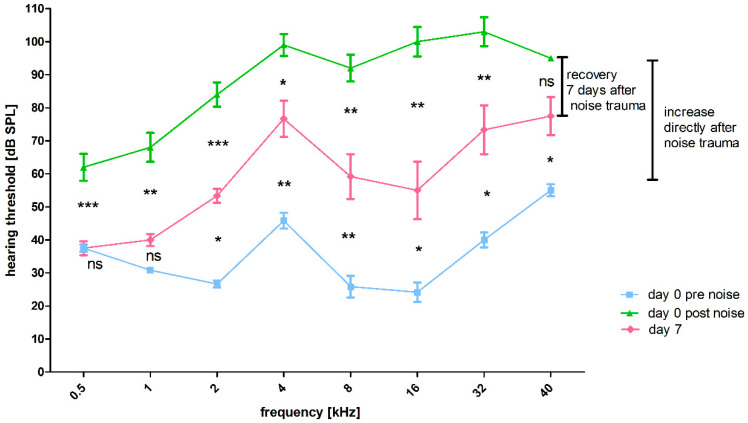
Effect of noise exposure on ABR pure tone thresholds in not implanted ears over time. Hearing thresholds of the right (not implanted) ears. Where the hearing threshold could not be identified up to the maximum sound stimulus level of 100 dB SPL (85 dB SPL for 40 kHz), the threshold was defined as 110 dB SPL or 95 dB SPL for 40 kHz. Directly after the noise insult, all thresholds increased significantly, except for 500 Hz and 1 kHz (blue line vs. green line, not illustrated). In addition, 7 days after the noise insult, there is significant frequency-specific recovery, except for 40 kHz (significances illustrated between the green and the red line). The remaining significant threshold shift from day 0 pre noise to day 7 was also frequency specific, except for 500 Hz, which showed a full recovery and 1 kHz (significances illustrated between the red and the blue graph). ns = not significant; * *p* ≤ 0.05; ** *p* ≤ 0.01; *** *p* ≤ 0.001.

The effect of the noise trauma is reflected in the difference in hearing thresholds before and 7 days after noise trauma in right (not implanted) ears. Compared to day 0 pre noise, there was a significant increase in frequency-specific thresholds 30 min after noise exposure (day 0 post noise), except for 500 Hz and 1 kHz (Figure 9, significances not illustrated). Compared to day 0 post noise exposure, significantly decreased frequency-specific thresholds were observed at day 7, except for 40 kHz (Figure 9, significances illustrated between the green and red line). This decrease in the frequency-specific hearing threshold from the period directly after trauma to day 7 after noise trauma proves that the applied noise results in a temporary threshold shift. Nevertheless, there was still a threshold shift in all frequencies other than 500 Hz and 1 kHz on day 7, in comparison to day 0 pre noise exposure (Figure 9, significances illustrated between blue and red line). Thus, in the period of one week only a partial recoveryoccurs.

#### 3.2.4. Effect of HDD Implantation and Noise Exposure on Frequency-Specific ABR Thresholds

**Figure 10:**
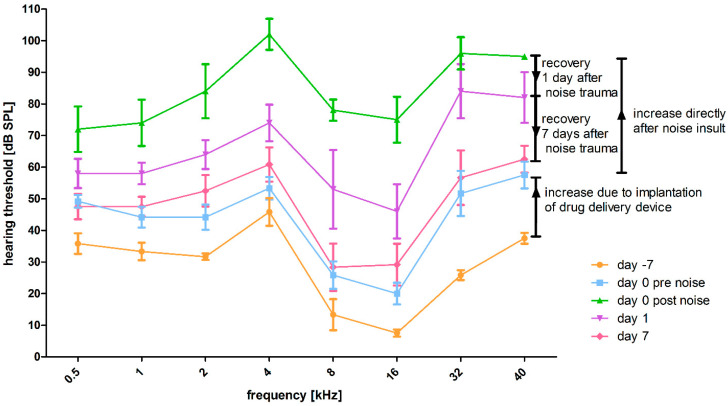
Effect of noise exposure on ABR pure tone thresholds in implanted ears over time. Mean frequency-specific hearing thresholds of the left (implanted and noise affected) ears are illustrated with standard deviation. Where the hearing threshold could not be identified up to the maximum sound stimulus level of 100 dB SPL (85 dB SPL for 40 kHz), the threshold was defined as 110 dB SPL or 95 dB SPL for 40 kHz. Day −7 is displayed in orange, day 0 before noise trauma in blue, day 0 post noise trauma in green, day 1 in purple and day 7 in pink.

The frequency-specific hearing thresholds over time of the left (implanted) and noise affected ears are descriptively illustrated in Figure 10. The thresholds of all frequencies increased due to implantation of the HDD and were additionally increased by noise insult. Directly one day after noise, recovery was observed, which progressed until observational day 7.

**Figure 11:**
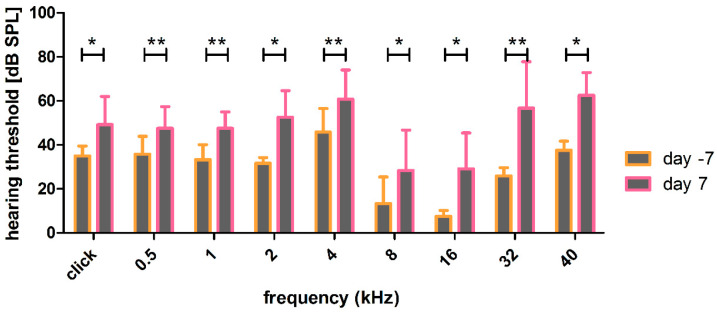
Effect of noise and implantation on hearing thresholds. The hearing thresholds of the implanted and noise traumatized ears show a significant, but frequency specific, increase over the whole time period. * *p* ≤ 0.05; ** *p* ≤ 0.01.

The combination of implantation and noise insult led to significantly increased thresholds over all frequencies, which were still detectable 7 days after noise trauma (Figure 11). The insult was less in the best frequency region around 8 to 16 kHz and more pronounced at high frequencies (32 and 40 kHz) and middle to low frequencies (0.5, 1, 2, 4 kHz).

#### 3.2.5. Effect of HDD Implantation on Noise Trauma

**Figure 12:**
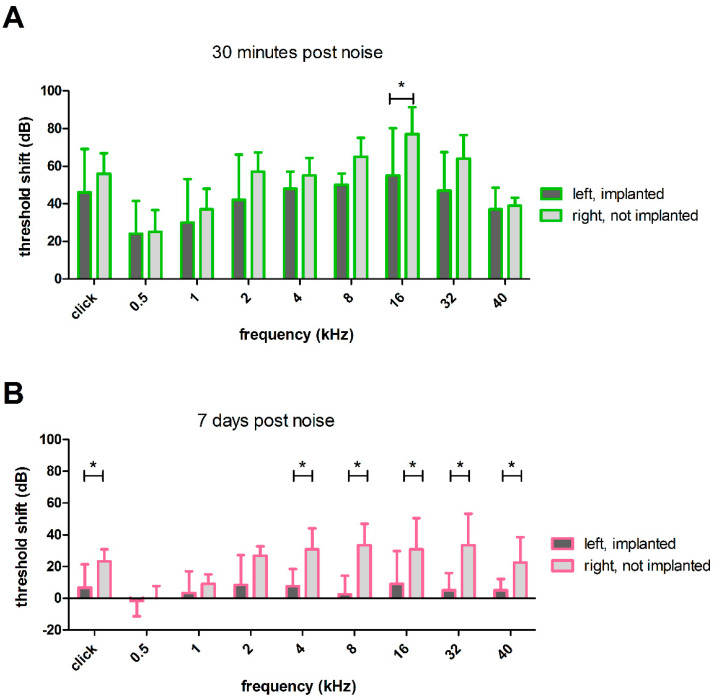
Noise-induced hearing threshold shifts of left (implanted) and right (not implanted) ears. (**A**) Directly after the noise trauma, there was a significant difference in the threshold shifts between left (implanted) and right (not implanted) ears at 16 kHz. (**B**) One week after the noise insult, there was frequency-specific recovery that led to a significant difference between the ears. Only 500 Hz, 1 kHz and 2 kHz showed no significant difference. Only significant differences are illustrated in A and B; * *p* ≤ 0.05.

The threshold shifts from the period before noise insult to directly after noise insult did not differ between the left (implanted) and right (not implanted) ears (Figure 12A), except for 16 kHz (55 ± 25.29 dB vs. 77 ± 14.4 dB, *p* ≤ 0.05; paired t-test}. However, 7 days after noise application, the threshold shift in left (implanted) and right (not implanted) ears differed significantly (Figure 12B), with implantation resulting in significantly lower threshold shifts in five out of eight frequencies compared to the right (not implanted) ears. For example, at 40 kHz, the mean threshold shift was only 5 ± 7.0 dB in left (implanted) ears, whereas in the right (not implanted) ears, the shift was significantly worse with 22 ± 16.0 dB (*p* ≤ 0.05; paired t-test). At 8 kHz, the mean threshold shift from day 0 before noise insult to day 7 after noise was 2 ± 11.7 dB in left (implanted) ears, and therefore significantly lower than in right (not implanted) ears, where the shift was 33 ± 13.6 dB (*p* ≤ 0.001; paired t-test). At 2 kHz, there was still a difference in threshold shifts between left (implanted) and right (not implanted) ears (8 ± 18.8 dB vs. 26 ± 6.0 dB, *p* ≤ 0.05; Wilcoxon signed-rank test), but at lower frequencies (1 kHz and 500 Hz), no differences were observed. In these frequency regions, the threshold shift of both groups was close to 0 with 3 ± 13 dB at 1kHz and −1 ± 9.8 dB at 500 Hz for left (implanted) ears and 9 ±−5.8 dB at 1 kHz and 0 ±−7.7 dB at 500 Hz for right (not implanted) ears, respectively.

### 3.3. µCT

**Figure 13:**
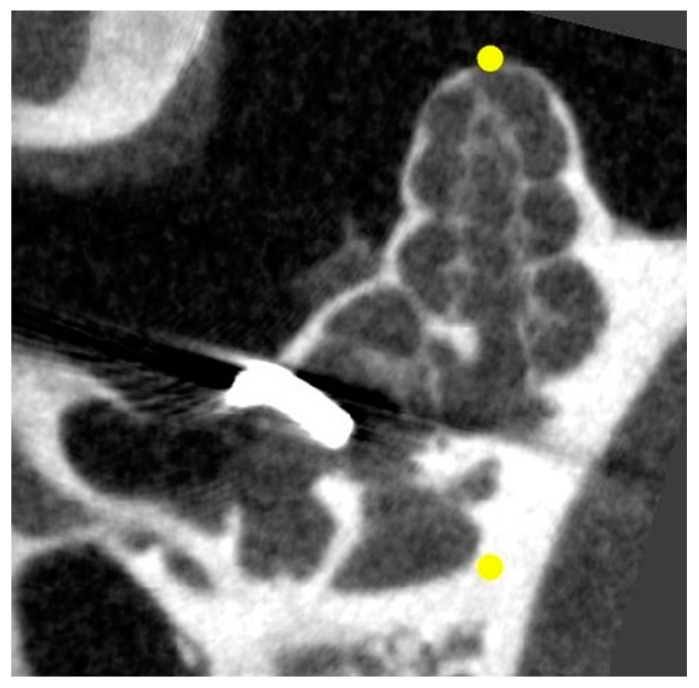
µCT scan of an implanted guinea pig cochlea. Analysis of the hook position was carried out by visual inspection of the DICOM data using COMET [42]. The yellow points indicate the rotation axis in the 3D reconstruction.

The microneedle can be visualized by µCT, allowing determination of the correct location of the hook (Figure 13). The analysis of day 1 µCT, as well as the day 7 µCT, revealed that the HDDs were correctly implanted in all animals and stayed in the RWN during the experimental period of 14 days.

## 4. Discussion

Numerous diseases cause cochlear dysfunction. Local pharmacotherapy could be a treatment option in the future. Prevention is always better than therapy; therefore, preventive drug treatment strategies should be applied before an insult affects cochlear health. To measure the biological effectiveness of a preventive local drug therapy, the application per se needs to be as less traumatic as possible. We are interested in identifying preventive substances against NIHL. For this purpose, we developed a model for pump-based drug delivery in normal hearing guinea pigs that received TTS-inducing noise trauma. Since drug delivery to the inner ear is challenging due to the blood–labyrinth-barrier and round window membrane permeability, delivery directly in the perilymphatic space using a pump-based system is the best way to investigate a drug’s effect in basic research. As soon as an effect is shown and the therapeutic dose is determined, the delivery method needs to be optimized for patients, as it would be too traumatic for the current pump-based delivery system to be applied for the treatment of inner ear pathologies. The application in human patients requires drug delivery in the form of systemic delivery or local delivery without opening the perilymphatic space. Therefore, the presented study aims to establish a drug delivery approach to release drugs in a known concentration and for a known duration into the inner ear for a basic research set-up.

A well-established method for chronic drug delivery to the inner ear is the use of osmotic minipumps [31,36,44]. They enable the delivery of substances directly into the perilymph, without the release kinetics being affected by a delivery matrix. In combination with a CI [32,33,34] or a tube [22,31,35,36], they transfer the fluids into the scala tympani through the round window or a cochleostomy [22,37]. To reduce the risk of surgery-related threshold shifts in mice, delivery of substances via the posterior semicircular canal is a common method [45], but in the guinea pig, the surgical access is difficult and the direct delivery to the cochlea is the standard method. Usually, osmotic pump-based inner ear delivery is performed in chemically deafened animals [32,33,35,37]; therefore, no surgery-related hearing threshold increase occurs. Animals from studies that deal with normal hearing were excluded due to their increased hearing threshold after implantation [22]. For establishing an animal model to investigate drug effects on noise affected ears, animals should have no impairment in hearing. Implantation of the HDD led to increased click and frequency-specific hearing thresholds, with significances in frequencies above 8 kHz (Figure 6A, Figure 8A, Figure 10). Hearing loss has been reported previously from round window interventions in guinea pigs [46]. Changes in the round window membrane stiffness following cochlear implantation are thought to be the reason for low-frequency hearing loss [47]. Nevertheless, the surgery-related threshold shift is smaller than that reported in other studies [38,48]. Additionally, Sale et al. reported that even the application of artificial perilymph on the intact round window membrane alone can cause hearing threshold increases [40]. This observation is accompanied by the negative effect of artificial perilymph described by Scheper et al. and Shepherd et al. [32,49]. They delivered artificial perilymph to chemically deafened animals and analyzed the survival of the spiral ganglion cells. In treated ears, the survival was reduced compared to the deafened untreated ears. Nevertheless, the use of artificial perilymph seems useful, since the osmolarity of the inner ear fluids affects the cochlear function [50]. The present study did not measure the frequency-specific hearing thresholds in the right (not implanted) ear before implantation. Therefore, the day −7 threshold of the later implanted sides represents normal hearing thresholds. When comparing the d0 threshold (pre noise) of right (not implanted) ears with this day −7 baseline threshold, a significant difference in hearing thresholds of left (implanted) and right (not implanted) ears on day 0, pre noise (Figure 8C), in some tested frequencies is observed. This leads to the partially not significant differences in hearing threshold above 4 kHz of left (implanted) and right (not implanted) ears on day 0 before noise exposure (Figure 8B). We hypothesize that a larger number of animals would lead to a significant difference in hearing thresholds of left (implanted) and right (not implanted) ears on day 0, pre noise (Figure 8B), in all tested frequencies.

However, it is also possible that there is no significant difference between left (implanted) and right (not implanted) ears in the higher frequencies, since a spread of inflammatory cells, for example, into the contralateral ear cannot be excluded. Unlike humans, guinea pigs have a large cochlear aqueduct that connects the perilymph spaces of both ears [51]. Additionally, the eustachian tube is known for contralateral spreading of substances injected intratympanically [52]. Since a part of the HDD is located in the cavum tympani, potential foreign body reactions could spread to the right (not implanted) ear, and therefore lead to an increased hearing threshold, making it difficult to detect a difference between the ears. Additionally, the changed electrode placement for ABR measurements after surgery may lead to increased hearing thresholds, and therefore may explain the differences in the hearing thresholds of day −7 of the left (implanted) ears and day 0 of the right (not implanted) ears (Figure 8C). However, we assume that the electrode position did not have a significant effect on the hearing thresholds.

We used a small microneedle that was shortened, bent and inserted 1.5 mm in the scala tympani. As the microneedle can be visualized using µCT, it is possible to perform a second surgery, if an animals’ implant is in an incorrect position. This follows the principle of the animal welfare law to reduce the number of animals used for scientific purposes. Care should be taken to ensure that the microcannula part of the implant ends in the middle ear cavity or can be completely covered with dental cement, while closing the osteotomy of the cavum tympani. Otherwise, it may lead to skin irritation or development of pressure, strain, or tension forces, changing the implant’s position.

As the study’s aim was to develop a NIHL animal model for preventive chronic drug delivery, the next step was to expose the animals to a noise insult. Many studies concerning noise trauma use an artificial noise trauma, such as a pure tone [19,29,53,54,55], an octave-band noise [22,25,45,56,57,58,59,60,61] or white noise [62,63,64,65]. In order to create a better transferability to humans by mimicking the natural fluctuations of the temporal signal envelope, we decided to use a modified orchestral piece. Since professional musicians have a higher risk of hearing disorders [66,67], using a modified orchestral piece may have a better relation to their experience. The noise exposure induced a temporary threshold shift in both left (implanted) (Figure 10) and right (not implanted) (Figure 9) ears. The end of the experiment was set at day 7 after the noise insult because a study by Hickman et al. showed a regeneration of affected synapses in the mature cochlea, with the largest change in the first week after noise exposure [68]. The drug delivery via osmotic pumps is continuous. As the aim was to investigate drug effects during long-term administration, there was a smooth transition from preventive to therapeutic effects. At day 7, the noise-induced threshold shifts of both ears differ significantly, except for 500 Hz, 1 kHz and 2 kHz (Figure 12A). The noise-induced threshold shift was determined as the difference between hearing thresholds after noise and day 0 pre noise. Due to the previous implantation, the left (implanted) ears, therefore, have a different starting level for the calculation. However, when the hearing thresholds were considered, a difference between left (implanted) and right (not implanted) ears was noticeable (Figure 9 and Figure 10). This finding is supported by a study by Eshraghi et al., where noise-exposed unimplanted cochleae showed higher threshold shifts than noise-exposed ones with an implant [69]. One possible explanation is that the left (implanted) ears suffer from conductive hearing loss due to the implantation. It is known that the hearing loss after cochlear implantation is mainly conductive [38] and the round window stiffness has an effect on residual hearing [47]. Thus, it is possible that the noise did not cause the same insult to the cochlear sensory cells as in the right (not implanted) ear. Additionally, the permanent flow of artificial perilymph produced by the osmotic pump may also influence the regeneration after noise trauma, by leading to an increased wash-out of toxic products, such as reactive oxygen species. This hypothesis is supported by the fact that the noise-induced threshold shift at day 0 did only differ significantly at 16 kHz between left (implanted) and right (not implanted) ears (Figure 12A). Nevertheless, it remains unclear how much AP reaches the apical turns of the cochlea. Even though there is no sealing of the round window membrane, and therefore the intracranial pressure might drive cerebrospinal fluid into the cochlea, drug delivery via osmotic pumps is successful [32]. Additionally, the guinea pig round window membrane heals quickly after microneedle perforation, usually within one week, with the highest healing rate within the first 48h [70]. Moreover, larger ruptures have been shown to heal spontaneously within two weeks [71]. Nevertheless, the exact fluid distribution in the cochlea remains unclear and should be investigated in future studies. One mechanism of NIHL is the generation of reactive oxygen species [72] and antioxidants can reduce NIHL [73]. The delivered artificial perilymph contains albumin, which is also known to have antioxidant effects [74]. Nevertheless, it does not make sense to omit the albumin. Albumin is needed for the aforementioned osmolarity and many potential active ingredients require a carrier protein for delivery. Without this carrier protein, the substances may get stuck at the catheter wall and may not reach the perilymph. Despite the better thresholds of the left (implanted) ears, the noise insult leads to a TTS that can be detected and it would be possible to test potentially preventive substances.

The shown tendency towards a beneficial effect of AP infusion, improving the recovery from noise, needs to be considered in future studies that investigate the beneficial effects of drugs on inner ear trauma in normal hearing animals. The potential protective effects of agents should lead to faster recovery compared with control ears implanted with an AP delivering pump or maybe an additive, or even synergistic effect, of drug and AP infusion. To elucidate which intervention, drug delivery approach and molecule cause which effect in the inner ear, the evaluation should include the not implanted (contralateral) ears and maybe other fluids, e.g., saline or AP without albumin, should be considered as control substances. However, as aforementioned, omitting the albumin may influence the osmolarity of the perilymph, and therefore may affect the endocochlear potential. Depending on which drug to be tested, albumin may be necessary as a carrier protein.

NIHL is a complex disease and many factors have to be considered when establishing a new animal model. For example, both the melatonin of pigmented animals [75] and the estradiol of females [76] show a protective effect, so the selection of the animals is an important factor. Moreover, the circadian rhythm should be considered when planning the experimental setup [77]. In order to reduce the animals’ stress, the presented study performed the noise trauma under general anesthesia. In contrast to other anesthetics [78], to our knowledge, no protective effect has been described for the anesthetic regime used in this study. Nevertheless, this is important to keep in mind when testing the noise paradigm in awake animals. In awake animals, lower sound levels are needed to induce the same acoustical trauma. Working with awake animals has another benefit, as body core temperature is more steady. Hypothermia is a generally known side effect of anesthesia and has also a protective effect against NIHL [79]. This fact reinforces the necessity to maintain the body temperature under anesthesia, e.g., with the use of a heating pad as we did in this study. When evaluating studies that use a NIHL, including its prevention or its treatment, all of these factors must be taken into consideration.

Even though the focus of the present study was on the development of an animal model for long-term application of preventive substances for NIHL, the presented HDD can also be used in other areas of hearing research. This includes all areas in which cochlear pharmacotherapy is investigated, such as the prevention of ototoxic drug effects, such as aminoglycosides or cisplatin, or the development of virus-mediated gene therapies.

## 5. Conclusions

The implantation of the HDD attached to an osmotic pump caused a moderate threshold shift that enabled us to further induce and detect a temporary noise-induced threshold shift. An exposure of 4 h to the audio file of the orchestral piece caused an increase in hearing threshold for all tested frequencies with a frequency-specific intensity. The threshold shift partially recovers within one week, with more pronounced recovery at the left (implanted) ear. This facilitates the investigation of the effect of drugs delivered prior to the noise insult via osmotic pumps to establish a preventive therapy against noise-induced TTS, an ailment with global importance.

## Figures and Tables

**Table 1:** Overview of the time points and scope of the ABR measurements for implanted and contralateral ears. Frequency-specific measurements included the frequencies 500 Hz, 1, 2, 4, 8, 16, 32, and 40 kHz.

Day	Implanted Ear	Not Implanted Ear
−7	Click, frequency specific	Click
0 pre noise	Click, frequency specific	Click, frequency specific
0 post noise	Click, frequency specific	Click, frequency specific
1	Click, frequency specific	Click
7	Click, frequency specific	Click, frequency specific

## Data Availability

The data presented in this study are available upon request from the corresponding author.

## Supplementary Materials

The supporting information can be downloaded at: https://www.mdpi.com/article/10.3390/biom12101427/s1, File S1: detailed information on the statistical tests performed.

## Appendix A

ABR measurement, drug delivery device implantation, noise trauma, µCT and euthanasia were performed under general anesthesia (intramuscular medetomidinhydrochloride (Dormilan^®^ 1 mg/mL, alvavet Tierarzneimittel GmbH, Germany) 0.2 mg/kg, midazolam (Midazolam 5 mg/mL, PANPHARMA GmbH, Germany) 1 mg/kg and fentanyl (Fentadon 50 µg/mL, Dechra Veterinary Products Deutschland GmbH, Germany) 0.025 mg/kg) with previous sedation (oral diazepam (Diazepam-ratiopharm^®^ 10 mg/mL Tropfen zum Einnehmen, ratiopharm GmbH, Germany) 4 mg/kg). To avoid eye desiccation, the animals received eye ointment (Bepanthen^®^, Bayer Vital GmbH, Germany). Areas to be incised were locally infiltrated with prilocaine (Xylonest 1%, Aspen Germany GmbH, Germany). Prior to anesthesia, animals received a probiotic (oral 0.5 g Bene-Bac^®^ (BENE-BAC^®^ Gel, Dechra Veterinary Products Deutschland GmbH, Germany) and on day 0 and day 1, additionally a prebiotic (oral Lactulose (Lactulose Albrecht^®^, Dechra Veterinary Products Deutschland GmbH, Germany) 615 mg/kg) to prevent indigestion due to the long anesthesia. They subcutaneously received 0.5 mg/kg atropine (Atropinsulfat B. Baun 0.5 mg/mL Injektionslösung, B. Braun Melsungen AG, Germany) or 0.02 mg/kg glycopyrronium bromide (Glycopyrroniumbromic Accord 200 Mikrogramm/mL Injektionslösung, Accord Healthcare B.V., Netherlands) at day 0 to reduce bronchial secretion and salivation, and 8 mL (4 mL at day 1 and 7) Ringer’s solution (Ringer-Lösung DELTAMEDICA, DELTAMEDICA GmbH, Germany), including 5% glucose (Glucose 40% B. Braun, Konzentrat zur Herstellung einer Infusionslösung, B. Braun Melsungen AG, Gemany) per 300 g body weight. The anesthesia was antagonized by injecting atipamezole (ATIPAZOLE 5 mg/mL, Prodivet pharmaceutics, Belgium), 1 mg/kg, flumazenil (Flumazenil-hameln 0.1 mg/mL, Hameln pharma GmbH, Germany) 0.1 mg/kg and naloxone (Naloxon Inresa 0.4 mg/mL, Inresa Arzneimittel GmbH, Germany) 0.03 mg/kg subcutaneously. To reduce pain and to prevent infections, the animals subcutaneously received 0.2 mg/kg meloxicam (Metacam^®^ 2 mg/mL, Boehringer Ingelheim Vetmedica GmbH, Germany) and 10 mg/kg enrofloxacin (Baytril^®^ 25 mg/mL, Elanco GmbH, Germany) at day −7. Additionally, the antagonization was conducted without naloxone to maintain the analgetic impact of fentanyl. Postoperative care included 3 days 0.2 mg/kg meloxicam (Metacam^®^ 0.5 mg/mL, Boehringer Ingelheimmedica GmbH, Germany) and 7 days 5 mg/kg enrofloxacin (Baytril^®^ 2.5%, Elanco GmbH, Germany) per os. In addition, animals received 0.5 g Bene-Bac^®^ the day after every anesthesia. Euthanasia was performed via intracardiac injection of no less than 300 mg/kg pentobarbital (Release^®^ 300 mg/mL, Wirtschaftsgenossenschaft deutscher Tierärzte eG, Germany).
