# Supplementary material for: Local Long-Term Inner Ear Drug Delivery in Normal Hearing Guinea Pig—An Animal Model to Develop Preventive Treatment for Noise-Induced Hearing Loss
Source: Biomolecules. 2022 Oct 5;12(10):1427. doi: 10.3390/biom12101427 (PMC9599559; doi:10.3390/biom12101427)
Supplement: Supplementary file 1 [file biomolecules-12-01427-s001.zip › biomolecules-1802883-supplementary.pdf]

“Local Long-term Inner Ear Drug Delivery in Normal Hearing Guinea Pig – an Animal Model to develop preventive treatment for noise-induced hearing loss“

Kathrin Malfeld, Peter Baumhoff, Holger A. Volk, Thomas Lenarz and Verena Scheper

Supplement S1: detailed information on the statistical tests performed

Figure 6A
all: paired t-test

Figure 6B
all: paired t-test

Figure 7
all: paired t-test

Figure 8A

comparison	test used
click	paired t-test
0.5 kHz	Wilcoxon signed-rank test
1 kHz	Wilcoxon signed-rank test
2 kHz	Wilcoxon signed-rank test
4 kHz	paired t-test
8 kHz	paired t-test
16 kHz	Wilcoxon signed-rank test
32 kHz	paired t-test
40 kHz	Wilcoxon signed-rank test

Figure 8B

comparison	test used
click	paired t-test
0.5 kHz	Wilcoxon signed-rank test
1 kHz	Wilcoxon signed-rank test
2 kHz	Wilcoxon signed-rank test
4 kHz	paired t-test
8 kHz	paired t-test
16 kHz	paired t-test
32 kHz	Wilcoxon signed-rank test
40 kHz	paired t-test

Figure 8C

comparison	test used
click	paired t-test
0.5 kHz	Wilcoxon signed-rank test
1 kHz	Wilcoxon signed-rank test
2 kHz	Wilcoxon signed-rank test
4 kHz	paired t-test
8 kHz	paired t-test
16 kHz	Wilcoxon signed-rank test
32 kHz	Wilcoxon signed-rank test
40 kHz	Wilcoxon signed-rank test

Figure 9

- **d0 pre noise vs. d0 post noise**

comparison	test used
0.5 kHz	Wilcoxon signed-rank test
1 kHz	Wilcoxon signed-rank test
2 kHz	Wilcoxon signed-rank test
4 kHz	paired t-test
8 kHz	paired t-test
16 kHz	paired t-test
32 kHz	Wilcoxon signed-rank test
40 kHz	Wilcoxon signed-rank test

- **d0 post noise vs. d7**

comparison	test used
0.5 kHz	paired t-test
1 kHz	paired t-test
2 kHz	paired t-test
4 kHz	paired t-test
8 kHz	paired t-test
16 kHz	paired t-test
32 kHz	paired t-test
40 kHz	Wilcoxon signed-rank test

- **d0 pre noise vs. d7**

comparison	test used
0.5 kHz	Wilcoxon signed-rank test
1 kHz	Wilcoxon signed-rank test
2 kHz	Wilcoxon signed-rank test
4 kHz	paired t-test
8 kHz	paired t-test
16 kHz	paired t-test
32 kHz	Wilcoxon signed-rank test
40 kHz	paired t-test

Figure 11

comparison	test used
click	paired t-test
0.5 kHz	paired t-test
1 kHz	paired t-test
2 kHz	Wilcoxon signed-rank test
4 kHz	paired t-test
8 kHz	paired t-test
16 kHz	Wilcoxon signed-rank test
32 kHz	paired t-test
40 kHz	Wilcoxon signed-rank test

Figure 12A

comparison	test used
click	paired t-test
0.5 kHz	paired t-test
1 kHz	Wilcoxon signed-rank test
2 kHz	paired t-test
4 kHz	paired t-test
8 kHz	paired t-test
16 kHz	paired t-test
32 kHz	paired t-test
40 kHz	paired t-test

Figure 12B

comparison	test used
click	paired t-test
0.5 kHz	paired t-test
1 kHz	paired t-test
2 kHz	Wilcoxon signed-rank test
4 kHz	paired t-test
8 kHz	paired t-test
16 kHz	Wilcoxon signed-rank test
32 kHz	paired t-test
40 kHz	paired t-test
